# De novo assembly and comparative transcriptome analysis of *Monilinia fructicola*, *Monilinia laxa* and *Monilinia fructigena*, the causal agents of brown rot on stone fruits

**DOI:** 10.1186/s12864-018-4817-4

**Published:** 2018-06-05

**Authors:** Rita M. De Miccolis Angelini, Domenico Abate, Caterina Rotolo, Donato Gerin, Stefania Pollastro, Francesco Faretra

**Affiliations:** Department of Soil, Plant and Food Sciences - Plant Pathology Section, University of Bari Aldo Moro, via Amendola 165/A, 70126 Bari, Italy

**Keywords:** Transcriptomics, Trinity assembly, RNA-Seq, Plant pathogens, Fungi, Stone fruit disease

## Abstract

**Background:**

Brown rots are important fungal diseases of stone and pome fruits. They are caused by several *Monilinia* species but *M. fructicola*, *M. laxa* and *M. fructigena* are the most common all over the world. Although they have been intensively studied, the availability of genomic and transcriptomic data in public databases is still scant. We sequenced, assembled and annotated the transcriptomes of the three pathogens using mRNA from germinating conidia and actively growing mycelia of two isolates of opposite mating types per each species for comparative transcriptome analyses.

**Results:**

Illumina sequencing was used to generate about 70 million of paired-end reads per species, that were de novo assembled in 33,861 contigs for *M. fructicola*, 31,103 for *M. laxa* and 28,890 for *M. fructigena*. Approximately, 50% of the assembled contigs had significant hits when blasted against the NCBI non-redundant protein database and top-hits results were represented by *Botrytis cinerea*, *Sclerotinia sclerotiorum* and *Sclerotinia borealis* proteins. More than 90% of the obtained sequences were complete, the percentage of duplications was always less than 14% and fragmented and missing transcripts less than 5%. Orthologous transcripts were identified by tBLASTn analysis using the *B. cinerea* proteome as reference. Comparative transcriptome analyses revealed 65 transcripts over-expressed (FC ≥ 8 and FDR ≤ 0.05) or unique in *M. fructicola*, 30 in *M. laxa* and 31 in *M. fructigena*. Transcripts were involved in processes affecting fungal development, diversity and host-pathogen interactions, such as plant cell wall-degrading and detoxifying enzymes, zinc finger transcription factors, MFS transporters, cell surface proteins, key enzymes in biosynthesis and metabolism of antibiotics and toxins, and transposable elements.

**Conclusions:**

This is the first large-scale reconstruction and annotation of the complete transcriptomes of *M. fructicola*, *M. laxa* and *M. fructigena* and the first comparative transcriptome analysis among the three pathogens revealing differentially expressed genes with potential important roles in metabolic and physiological processes related to fungal morphogenesis and development, diversity and pathogenesis which need further investigations. We believe that the data obtained represent a cornerstone for research aimed at improving knowledge on the population biology, physiology and plant-pathogen interactions of these important phytopathogenic fungi.

**Electronic supplementary material:**

The online version of this article (10.1186/s12864-018-4817-4) contains supplementary material, which is available to authorized users.

## Background

Brown rots are major diseases of stone and pome fruits that can cause severe yield losses during both field production and post-harvest processing [1]. Several species of *Monilinia* genus are responsible for the diseases, but *Monilinia fructicola* (G. Winter) Honey (MFRC), *Monilinia laxa* (Aderh. & Ruhland) Honey (MLAX) and *Monilinia fructigena* Honey (MFRG) are the most common species all over the world [1–3]. MLAX and MFRG have been the only ones present in Europe until 2001 when MFRC has been introduced, rapidly spread and become prevalent on the former indigenous species [4, 5]. Some phenotypic differences in fitness parameters such as growth rate in response to temperature, fungicide sensitivity and virulence have been recorded among the three species [5–7] but the reasons underlying its successfulness have not yet well clarified.

Although the three fungi have been deeply studied, the availability of genomic and transcriptomic data in public databases is still scant. There are only few published studies on the genetic variation in *Monilinia* species using different molecular markers, i.e. internal transcribed spacer (ITS) [8, 9], random amplified polymorphic DNA (RAPD) [6], simple sequence repeat (SSRs) and inter SSR (ISSR) [10, 11], and the characterization of specific genes, such as genes responsible for fungicide resistance [12] or pathogenicity [13].

In this study, we used Illumina sequencing of mRNA from conidia and mycelia of MFRC, MLAX and MFRG to obtain a de novo Trinity-based assembly of their transcriptomes. Orthologous transcripts were identified and compared to detect those apparently unique or differentially expressed in each species.

## Methods

### Samples

Two isolates of opposite mating type per each species, determined by PCR-based mating type assays [14], collected from naturally infected fruits sampled in orchards located in Italy, were used in this study (Table 1). The isolates were grown under different conditions to obtain comprehensive transcriptomes: i) mycelium grown at 21 ± 1 °C on cellophane disks overlaid on potato dextrose agar (infusion from 200 g peeled and sliced potatoes kept at 60 °C for 1 h, 20 g dextrose, adjusted at pH 6.5, 20 g agar Oxoid No. 3, per litre) in the dark for 4 days; ii) mycelium grown as above but in the dark for 2 days and then exposed 12 h per day to a combination of 2 daylight (Osram L36 W/20) and 2 near-UV (Osram, L36/73) lamps for 2 days; iii) conidia (1 × 10^5^ mL^− 1^) germinating in malt extract medium (20 g malt extract Oxoid, per litre) after 14 h at 24 ± 1 °C in darkness under shaking (120 rpm).

**Table 1:** *Monilinia* isolates and experimental conditions

Species	Isolate	Mating type	Origin		Sample ID ^¥^
			Host plant	Location	
*Monilinia fructicola*	Mfrc123	*MAT1–1*	Cherry	Puglia	Mfrc123 D
					Mfrc123 L
					Mfrc123 C
	Mfrc78	*MAT1–2*	Cherry	Campania	Mfrc78 D
					Mfrc78 L
					Mfrc78 C
*Monilinia laxa*	Mlax316	*MAT1–1*	Cherry	Puglia	Mlax316 D
					Mlax316 L
					Mlax316 C
	Mlax297	*MAT1–2*	Cherry	Puglia	Mlax297 D
					Mlax297 L
					Mlax297 C
*Monilinia fructigena*	Mfrg269	*MAT1–1*	Plum	Basilicata	Mfrg269 D
					Mfrg269 L
					Mfrg269 C
	Mfrg344	*MAT1–2*	Pear	Emilia-Romagna	Mfrg344 D
					Mfrg344 L
					Mfrg344 C

^¥^D = mycelium grown in the dark for 4 days; L = mycelium grown in the dark for 2 days and then exposed to light for 2 days; C = germinating conidia

### RNA extraction, library preparation and sequencing

Total RNA was extracted from a total of 18 samples (6 per species) made up by 100 mg of mycelium or germinated conidia with the RNeasy Plant Mini Kit (Qiagen, Milan, Italy), following the manufacturer’s protocol. cDNA libraries were prepared from 4 μg of total RNA using the TruSeq RNA Sample Preparation Kit v2 (Illumina, Inc., San Diego, CA, USA) and validated according to Illumina’s low-throughput protocol. The protocol was adjusted to obtain an average library size of about 400 bp (insert length 130–290 bp), by reducing RNA fragmentation time to 2 min at 94 °C. Sequencing was carried out on an Illumina HiScanSQ platform using TruSeq SBS kit v3 (Illumina, Inc.) to obtain paired-end reads, 92 nt in length. RNA and DNA quantity and quality were determined with a Nanodrop 2000 spectrophotometer (Thermo Fisher Scientific Inc., Wilmington, DE, USA) and a Bioanalyzer 2100 (Agilent Technologies, Santa Clara, CA, USA). After removing indexed adapters, reads from each library were filtered for quality score (QS ≥ 30) using CASAVA v1.8 software (Illumina, Inc.).

### Sequencing read quality and trimming

Reads were analysed for quality statistics, nucleotide distribution and redundancy using FastX-tools (http://hannonlab.cshl.edu/fastx_toolkit), and trimmed with Trimmomatic 0.36 (http://www.usadellab.org/cms/index.php?page=trimmomatic) setting the parameters as follows: i) LEADING and TRAILING = 3, removing bases from the two ends of the reads if below a threshold quality of 3; ii) SLIDING WINDOW = 4:2, cutting the reads when the average quality within the window composed of 4 bases falls below a threshold equal to 2; iii) MINLEN = 50, removing the reads shorter than 50 bp [15].

### Transcriptome de novo assembly

Trinity software v.2.1.1 (https://github.com/trinityrnaseq/trinityrnaseq/wiki) was used for the de novo assembly of the transcriptomes using together sequencing data from the six libraries (2 isolates and 3 growing conditions) from each species (Table 1). Default assembly parameters of Trinity were used, with the addition of the “--jaccard_clip” function because a high gene density with overlapping of UnTranslated Region (UTR) was expected [16].

### Functional annotation

The annotation of the putative transcripts obtained from each assembly was performed using local BLAST+ 2.3.0 (ftp://ftp.ncbi.nlm.nih.gov/blast/executables/blast+/) [17] and Blast2GO PRO v4.0.7 [18]. BLASTx analysis was carried out by searching against the NCBI non-redundant protein database (downloaded 22 November 2016) and setting E-value cut off at 10^− 3^. Blast2GO PRO was used to predict Gene Ontology (GO) terms, to assign the assembled sequences to the Kyoto Encyclopedia of Genes and Genomes (KEGG) pathways, and to analyse protein domains using the InterProScan tool. Blast2GO annotation search was conducted with 10^− 6^ as the E-value hit filter, 55 as the annotation cut-off and 5 as the GO weight; no HSP-hit coverage cut-off was considered. Moreover, all GO terms retrieved via InterProScan analysis were added and used to validate GO annotations; further enhancement of GO terms was conducted with Annotation Expander (ANNEX).

### Transcriptome quality assessment

For the first quality assessment, trimmed reads were realigned on the assembled transcriptomes using CLC Genomics Workbench v.7.0.3 (CLC bio, Aarhus, Denmark), setting the parameters as follows: i) mismatch cost = 2; ii) insertion cost = 3; iii) deletion cost = 3; iv) length fraction = 0.8; v) similarity fraction = 0.8.

The Trinity script analyze_blastPlus_topHit_coverage.pl (https://github.com/trinityrnaseq/trinityrnaseq/wiki/Counting-Full-Length-Trinity-Transcripts) was launched to determine the number of full-length or nearly full-length transcripts using BLAST+ with an E-value cut off 10^− 20^ and a protein database built from the proteome of *B. cinerea* (ASM83294v1; http://fungi.ensembl.org/Botrytis_cinerea/Info/Index, downloaded 10 April 2016), used as the closest related organism.

Putative Open Reading Frames (ORFs) within transcript sequences were predicted with TransDecoder v2.1 (http://transdecoder.github.io) and transcriptome completeness was assessed using BUSCO v1.2 (Benchmarking Universal Single-Copy Orthologs; http://busco.ezlab.org) [19] on the ground of 1438 conserved fungal orthologs (downloaded 21 October 2016).

### Comparative transcriptome analysis

tBLASTn search was used to identify orthologous transcripts in the three *Monilinia* species. In detail, *Botrytis cinerea* B05.10 proteins (ASM83294v1) integrated with the mitochondrial proteins (ftp://ftp.broadinstitute.org/pub/annotation/fungi/botrytis_cinerea/genomes/botrytis_cinerea_b05.10_mito, downloaded 10 April 2016) were queried against the assembled transcriptomes of MFRC, MLAX and MFRG, to identify homologous sequences. tBLASTn was run using CLC Genomics Workbench with a threshold E-value< 10^− 3^. Reads were then mapped on the selected transcripts and the unmapped reads re-aligned on the complete Trinity transcriptomes, to retrieve *Monilinia* transcripts with not homologs in the *B. cinerea* proteome; all transcripts with counted reads > 50 and significant matches to fungal proteins in BLASTx search were included for further analysis. Homology between the putative *Monilinia* orthologs was assessed through BLASTn pairwise alignments in all possible combinations (MFRC vs MLAX; MFRC vs MFRG; and MFRG vs MLAX) with the threshold parameter of E-value< 10^− 10^.

Reads from each isolate per species were mapped on the identified set of orthologous transcripts using CLC Genomics Workbench with the previously reported alignment parameter setting. The values of gene expression were measured in reads per Kb of transcript per million mapped reads (RPKM). The data from the three growing conditions for each isolate were pooled and used as two biological replicates per each species. Fold Change (FC) was calculated comparing the RPKM values in all pairwise combinations between the three fungal species. False Discovery Rate (FDR) was determined using the edgeR Bioconductor package [20] and transcripts with FC > |8| and FDR ≤ 0.05 in both the comparisons of each species with the other two were considered as differentially expressed transcripts (DETs) and submitted to functional analysis. For an accurate comparison among the species, we removed from the identified DET sets: i) incorrectly associated transcripts revealed by pairwise BLASTn alignments, including those with doubtful RPKM values due to multiple isoforms; and ii) chimeric transcripts identified by BLASTx, i.e. assembled sequences derived from two or more adjacent transcribed genes [21].

WEGO was used to perform functional classification of Trinity unigenes based on the GO annotation and compare the overall distribution of gene functions in the three species using the Pearson Chi-Square test [22]. All unigenes from each transcriptome were also submitted to search against the EggNog (Evolutionary genealogy of genes: Non-supervised Orthologous Groups) database, integrated in the Blast2GO pipeline, to predict and classify gene functions based on sequence similarity within clusters of orthologous groups (OGs) [23].

## Results

### Sequencing and transcriptome de novo assembly

Illumina mRNA sequencing from germinating conidia and actively growing mycelia of two isolates of opposite mating type per each species generated a total of 19.5 Gb, more than 6 Gb per species corresponding to about 70 million of paired-end reads (Additional file 1: Table S1).

After trimming of low quality reads, less than 1% of input paired-end reads were discarded, and the remaining reads used for de novo assembly. The most relevant data are in Table 2. Overall, Trinity assembly generated 33,861 contigs for MFRC, 31,103 for MLAX and 28,890 for MFRG corresponding to putative transcripts, including isoforms. The number of unigenes assembled by Trinity exceeded the expected number of protein coding genes, likely due to fragmented transcripts, i.e. unigenes representing the same transcript that could not be assembled because containing a gap.

**Table 2:** Statistics of Trinity assembly

	*Monilinia fructicola*	*Monilinia laxa*	*Monilinia fructigena*
Number of transcripts	33,861	31,103	28,890
Number of unigenes	27,692	27,334	25,781
Average transcript length (bp)	1545	1334	1199
Transcript N50	2799	2397	2189
Maximum length (bp)	13,605	15,609	15,798
Total assembled bases (Mb)	52.3	41.6	34.6
GC content (%)	45	44	44

### Quality assessment of de novo assembly

More than 80% of the paired-end reads used for the assembly successfully mapped to the respective assembled transcriptome used as reference (Additional file 1: Table S2).

About 8000 transcripts of each transcriptome showed homology with *B. cinerea*. Most of them (84% for MFRC, 83% for MLAX, 79% for MFRG) displayed a coverage higher than 70% of the protein length. TransDecoder identified 23,738, 18,756 and 16,823 candidate ORFs in the MFRC, MLAX and MFRG transcriptomes and 76.4, 72.7 and 65.5%, in the order, were complete.

Data obtained from transcriptome completeness analysis by BUSCO, based only on conserved fungal orthologs, showed that over 90% of the assembled transcripts for the three *Monilinia* species were complete and that the percentage of duplicated transcripts was always less than 14%. Less than 10% of transcripts were fragmented or missing (Table 3).

**Table 3:** Results of BUSCO analysis on the assembled transcriptomes

Species	Orthologs (N°) ^¥^				
	Complete			Fragmented	Missing
	Total	Single-copy	Duplicated		
*Monilinia fructicola*	1317 (91%)	1110 (77%)	207 (14%)	51 (4%)	70 (5%)
*Monilinia laxa*	1327 (92%)	1167 (81%)	160 (11%)	40 (3%)	71 (5%)
*Monilinia fructigena*	1303 (90%)	1146 (80%)	157 (10%)	67 (5%)	68 (5%)

^¥^ Percentages refer to the BUSCO dataset including 1438 conserved fungal orthologs (http://busco.ezlab.org/v1)

### Functional annotation

**Fig. 1:**
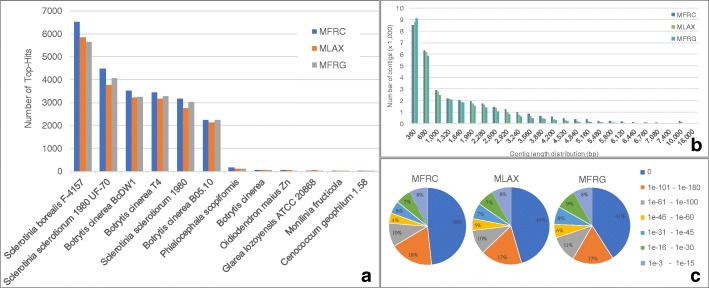
Top-hit species (**a**), contig length (**b**) and E-value (**c**) distributions in the transcriptomes of *Monilinia fructicola* (MFRC), *Monilinia laxa* (MLAX) and *Monilinia fructigena* (MFRG)

More than 50% of the assembled contigs had a significant hit in BLASTx search (Table 4). The top-hits by species distribution analysis showed the highest similarities with *B. cinerea* (strains BcDW1, T4 or B05.10), *Sclerotinia sclerotiorum* (1980 UF-70) and *Sclerotinia borealis* (F-4157) (Fig. 1a). The E-value distribution of the top blast hits showed about 50% of hits with an E-value equal to zero and about 70% with E-values ranging from 0 to 1e-61 (Fig. 1c). Sequences with no significant hits were mostly short fragments (Fig. 1b) or non-coding RNA sequences. The GO search assigned 18,839 GO terms, including the three main categories of biological process, molecular function and cellular component, to 13,538 contigs in MFRC, 16,462 GO terms to 12,082 contigs in MLAX, and 16,776 GO terms to 12,238 contigs in MFRG.

**Table 4:** Results of functional analysis of transcriptomes

Numbers	*Monilinia fructicola*	*Monilinia laxa*	*Monilinia fructigena*
Total transcripts	33,861	31,103	28,890
No Blast Hits	15,382	14,304	12,243
With Blast Hits	2902	2928	2670
With Mapping	2055	1814	1580
With GO Annotation	13,522	12,057	12,397
Transcripts with significant hit (%)	54.5	54.0	57.6

**Fig. 2:**
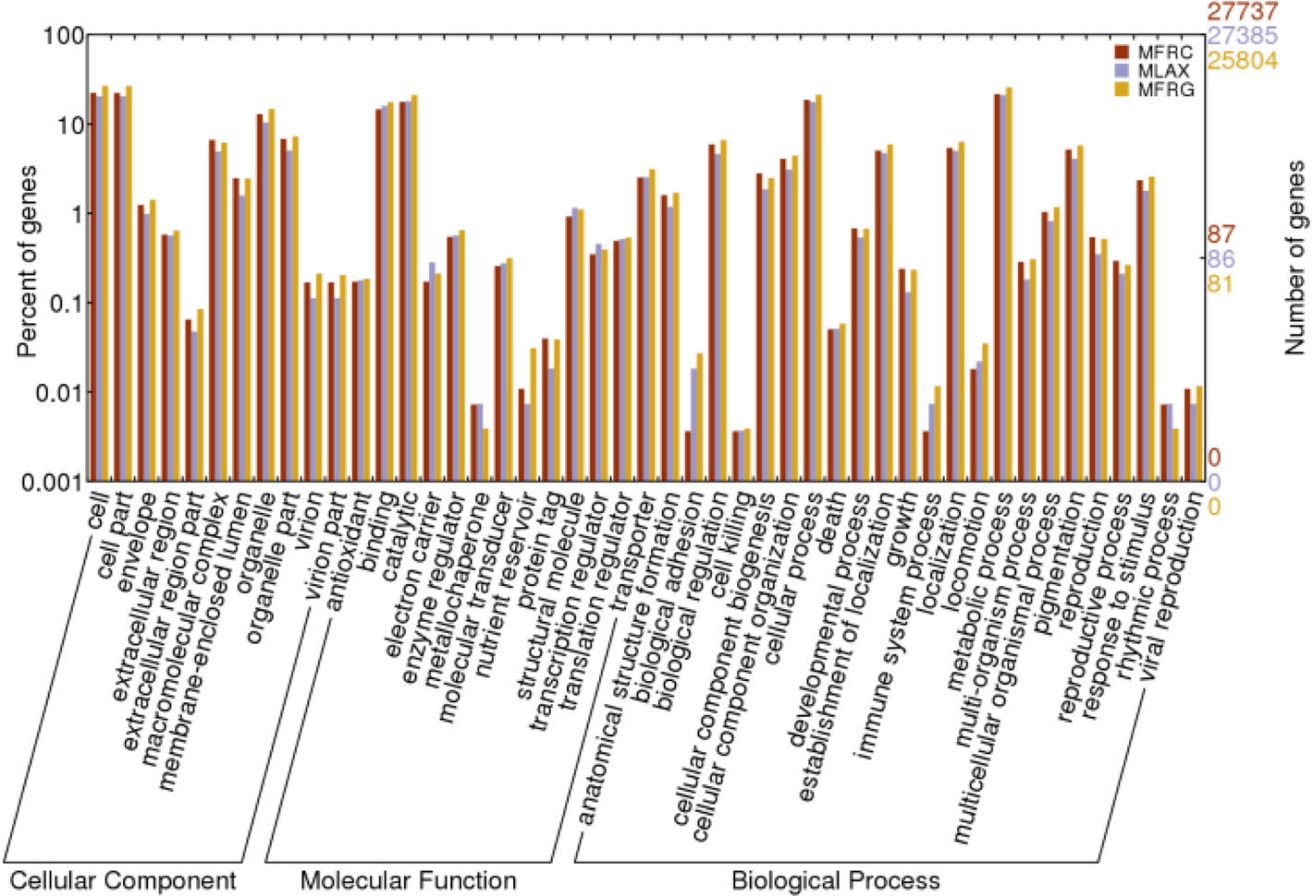
Frequency distribution of Gene Ontology (GO) terms grouped into the main functional categories of cellular component, molecular function and biological process. The right y-axis indicates the number of unigenes per category. The left y-axis indicates the percentage of a specific category of unigenes in the main category

In the three transcriptomes, more than 80% of the unigenes with a significant hit were associated to at least one GO term, and the GO terms were functionally classified and plotted. The three transcriptomes were very similar in their profiles of unigene distribution in functional categories (Fig. 2). Many unigenes were assigned to cellular and metabolic processes and to biological processes related to morphogenesis, localization, pigmentation, development and growth, reproduction, response to stimulus, multi-organism and multicellular organismal processes. In the molecular function category, binding and catalytic activities represented the majority followed by transporters, structural molecules and enzymes, transcription and translation regulation. In the cellular component category, cell, cell part, organelle and macromolecular complex were prevalently represented.

**Fig. 3:**
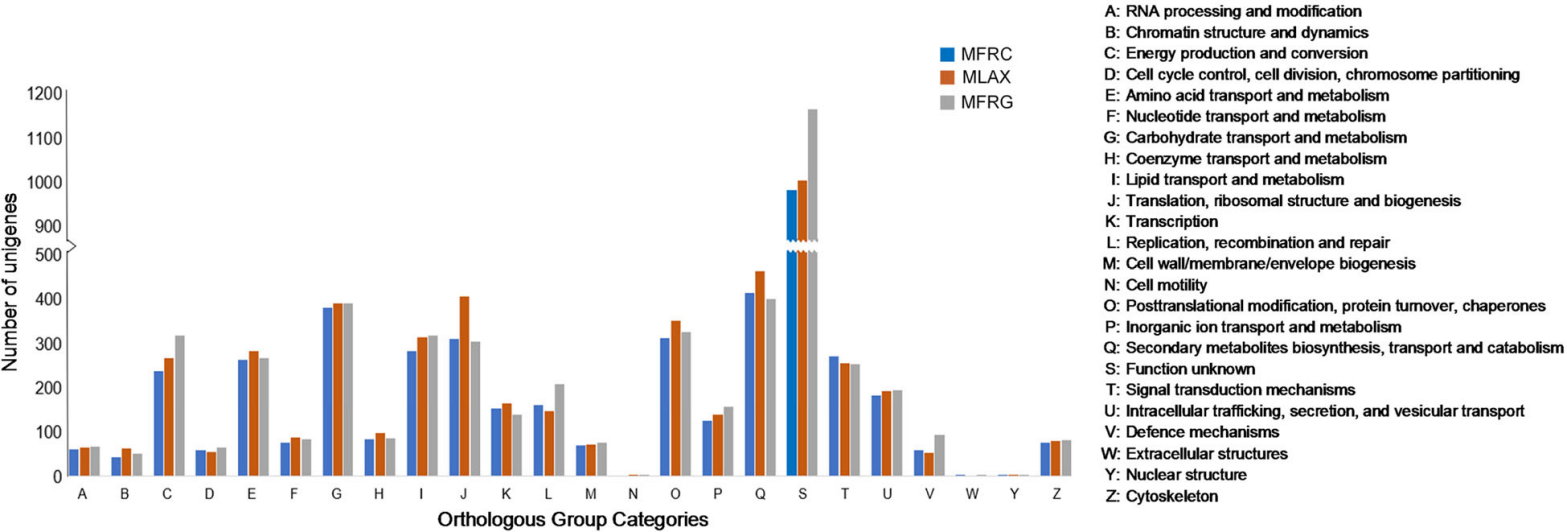
Distribution of *Monilinia fructicola* (MFRC), *Monilinia laxa* (MLAX) and *Monilinia fructigena* (MFRG) unigenes in clusters of OGs

In addition, BLAST and GO mapping results were used to assign unigenes to clusters of OGs using the EggNog database. In total 2536 (MFRC), 2734 (MLAX) and 2753 (MFRG) unigenes (17–23% of the genes with BLAST hits) were assigned to 24 functional categories of clusters of OGs (Fig. 3). The largest group was represented by the cluster of unigenes with “function unknown” (37–43%), followed by “secondary metabolites biosynthesis, transport and catabolism” (14–17%), “carbohydrate transport and metabolism” (14–15%), “translation, ribosomal structure and biogenesis” (11–15%), “posttranslational modification, protein turnover, chaperones” (12–13%), “lipid transport and metabolism” (11%), “energy production and conversion” (9–11%), “signal transduction mechanisms” (9–11%), “amino acid transport and metabolism” (10%). Clusters such as “cell motility”, “extracellular structures” and “nuclear structure” were poorly represented (< 0.1%) or absent. The number of unigenes enclosed in the OG clusters “function unknown” in MFRG and “translation, ribosomal structure and biogenesis” in MLAX were higher as compared to the other two species.

### Comparative transcriptome analysis

Ten-thousand four-hundred and fifty-two putative orthologous transcripts were identified among the three *Monilinia* species. In detail, 10,045 orthologs were identified by tBLASTn analysis using the proteome of *B. cinerea* as reference, whereas 407 transcripts were specific for at least one of the *Monilinia* species and had not homologous sequences in *B. cinerea* proteins.

**Fig. 4:**
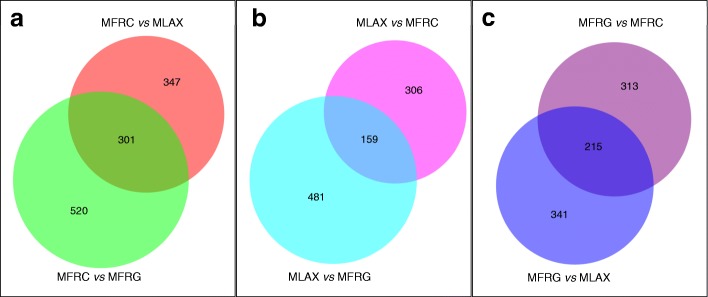
Venn diagrams of transcripts exclusive for or over-expressed (FC ≥ 8; FDR ≤ 0.05) in *Monilinia fructicola* (MFRC; **a**), *Monilinia laxa* (MLAX; **b**) and *Monilinia fructigena* (MFRG; **c**) as compared to the other two species

**Fig. 5:**
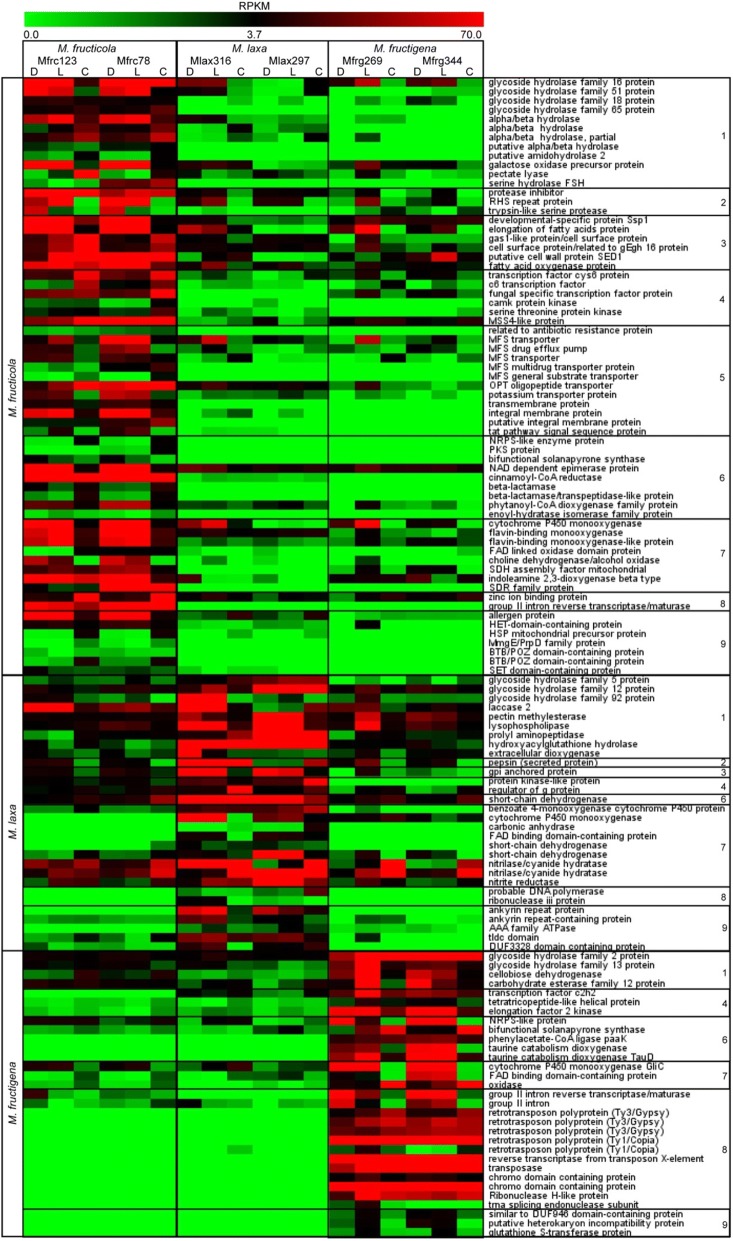
Heat map showing DET expression profiles in samples of mycelium grown in darkness (D) or exposed to light (L) and germinating conidia (C) from two isolates per species. DETS are grouped in functional classes: 1) hydrolytic and carbohydrate-active enzymes; 2) effector and secreted proteins; 3) morphogenesis and development; 4) regulation and signalling; 5) membrane proteins and transport; 6) secondary metabolite biosynthesis; 7) oxidation-reduction processes; 8) nucleic acid modification and metabolism; and 9) miscellaneous

Differential expression analysis of orthologs was carried out by comparing RPKM values in each species versus the other two. We identified 301 exclusive or over-expressed orthologs (FC ≥ 8; FDR ≤ 0.05) in MFRC, 159 in MLAX and 215 in MFRG (Fig. 4). Transcripts with uninformative BLAST hits (~ 45%, for MFRC and MLAX, and 22% for MFRG) were discharged. Finally, we identified 65 DETs significantly over-expressed or exclusive for MFRC, 30 in MLAX and 31 in MFRG, that were categorized into nine major functional groups: 1) hydrolytic and carbohydrate-active enzymes, 2) effector and secreted proteins; 3) morphogenesis and development; 4) regulation and signalling; 5) membrane proteins and transport; 6) secondary metabolite biosynthesis; 7) oxidation-reduction processes; 8) nucleic acid modification and metabolism; and 9) miscellaneous (Table 5). Heat-map representation of expression profiles of the DETs from the two isolates per species under the different growing conditions confirmed the diversity among the species revealed by mapping pooled reads of each isolate and showed differences between germinating conidia and mycelium samples that are not analysed in the present paper (Fig. 5).

**Table 5:** Differentially expressed transcripts (DETs) identified in *Monilinia fructicola* (MFRC), *Monilinia laxa* (MLAX) and *Monilinia fructigena* (MFRG)

Transcript ID	*Botrytis cinerea* protein ID^¥^	MFRC		MLAX		MFRG		Expression values			Putative protein function (BLASTx)
		Contig size (bp)	E-value (tBLASTn)	Contig size (bp)	E-value (tBLASTn)	Contig size (bp)	E-value (tBLASTn)	(RPKM)			
								MFRC	MLAX	MFRG	
Hydrolytic and carbohydrate-active enzymes											
T9687	Bcin12g01530.1	2023	0.0E + 00	2275	0.0E + 00	2093	0.0E + 00	**209.2**	12.9	18.3	glycoside hydrolase family 16 protein
T9618	Bcin09g04440.1	2342	0.0E + 00	1399	0.0E + 00	247	8.0E-43	**45.8**	3.7	0.4	glycoside hydrolase family 51 protein
T10205	Bcin03g01930.1	3806	6.4E-15	233	–	619	1.4E-30	**9.9**	0.3	1.0	glycoside hydrolase family 18 protein
T10086	NH	745	–	–	–	–	–	**12.9**	–	–	glycoside hydrolase family 65 protein
T309	Bcin07g02650.1	1555	0.0E + 00	1443	0.0E + 00	579	9.9E-102	2.4	**23.3**	1.2	glycoside hydrolase family 5 protein
T288	Bcin13g02320.1	1628	1.4E-118	1166	1.2E-120	1306	4.5E-120	9.1	**95.8**	11.5	glycoside hydrolase family 12 protein
T310	Bcin06g00580.1	3397	0.0E + 00	2763	0.0E + 00	1871	0.0E + 00	3.4	**40.2**	5.0	glycoside hydrolase family 92 protein
T6223	Bcin05g01040.1	3259	0.0E + 00	3361	0.0E + 00	3461	0.0E + 00	7.3	6.2	**137.4**	glycoside hydrolase family 2 protein
T7115	Bcin02g01420.1	1898	0.0E + 00	1148	9.7E-175	1966	0.0E + 00	5.1	3.6	**46.0**	glycoside hydrolase family 13 protein
T9625	Bcin02g05500.1	1569	0.0E + 00	1814	0.0E + 00	221	1.7E-09	**56.7**	4.4	0.5	α/β hydrolase
T9489	NH	2787	–	1261	–	–	–	**15.0**	1.8	–	α/β hydrolase
T9732	NH	535	–	531	–	361	–	**29.0**	1.5	0.6	α/β hydrolase, partial
T10098	NH	1969	–	–	–	–	–	**3.0**	–	–	putative α/β hydrolase
T10089	NH	2310	–	–	–	–	–	**2.3**	–	–	putative amidohydrolase 2
T9679	Bcin13g05710.1	4801	0.0E + 00	4584	0.0E + 00	3012	0.0E + 00	**113.9**	7.2	9.3	galactose oxidase precursor protein
T9581	Bcin10g05620.1	1278	2.3E-100	1211	6.7E-100	1225	1.4E-101	**32.1**	3.1	2.4	pectate lyase
T9586	Bcin14g00570.1	903	2.8E-61	–	–	–	–	**17.9**	–	–	serine hydrolase FSH
T53	Bcin01g07950.1	2112	0.0E + 00	2475	0.0E + 00	2303	0.0E + 00	3.1	**198.8**	11.8	laccase 2
T99	Bcin03g03830.1	1683	6.8E-179	1640	1.9E-177	1352	0.0E + 00	7.4	**245.0**	23.9	pectin methylesterase
T163	Bcin01g02320.1	3743	0.0E + 00	4284	0.0E + 00	2008	0.0E + 00	11.3	**228.3**	26.8	lysophospholipase
T181	Bcin12g04910.1	7278	1.3E-108	1847	2.6E-113	1546	6.7E-101	4.1	**74.9**	4.6	prolyl aminopeptidase
T96	Bcin03g05490.1	1965	3.2E-175	1229	1.3E-176	1544	5.7E-174	2.9	**95.1**	3.1	hydroxyacylglutathione hydrolase
T240	Bcin02g00018.1	1916	2.5E-93	1436	4.2E-94	1694	1.1E-95	3.1	**38.6**	4.5	extracellular dioxygenase
T9172	NH	3183	–	1985	–	3442	–	6.7	1.4	**61.3**	cellobiose dehydrogenase
T8110	Bcin02g07100.1	3717	1.7E-144	1579	5.5E-151	1028	2.2E-152	9.1	4.5	**112.2**	carbohydrate esterase family 12 protein
Effector and secreted proteins											
T9637	Bcin05g01800.1	1507	3.7E-87	1157	6.0E-71	1101	7.2E-73	**74.7**	5.8	1.9	protease inhibitor
T9520	Bcin14g04460.1	5479	0.0E + 00	5131	0.0E + 00	2153	0.0E + 00	**104.0**	11.6	9.2	RHS repeat protein
T9914	Bcin11g02900.1	1018	3.6E-158	365	8.9E-65	997	6.0E-91	**20.2**	0.1	2.0	trypsin-like serine protease
T144	Bcin03g03300.1	2948	5.4E-98	1189	1.5E-126	2152	7.7E-69	6.5	**144.7**	9.3	pepsin (secreted protein)
Morphogenesis and development											
T9903	NH	1255	–	1904	–	1965	–	**593.7**	4.0	18.1	developmental-specific protein Ssp1
T9722	Bcin15g04790.1	1315	2.2E-177	1359	4.5E-176	1303	2.4E-180	**315.4**	16.5	31.1	elongation of fatty acid protein
T9697	Bcin08g05540.1	1612	2.6E-124	2603	2.7E-120	1525	3.4E-123	**89.4**	5.6	4.5	gas1-like protein/cell surface protein
T9572	Bcin14g04260.1	4771	2.8E-157	2666	3.4E-163	1333	1.5E-165	**70.1**	7.0	7.6	cell surface protein/related to gEgh 16 protein
T9843	Bcin15g03000.1	1843	4.2E-53	1782	8.1E-46	2404	1.1E-56	**186.9**	3.2	11.8	putative cell wall protein SED1
T9480	Bcin02g03060.1	3163	0.0E + 00	3630	0.0E + 00	1999	0.0E + 00	**85.8**	10.6	5.9	fatty acid oxygenase protein
T205	Bcin12g05860.1	1706	3.6E-159	1271	2.4E-158	1141	3.1E-157	7.5	**114.0**	3.4	gpi anchored protein
Regulation and signalling											
T9760	Bcin12g01420.1	2515	0.0E + 00	2372	0.0E + 00	1730	0.0E + 00	**38.5**	1.8	4.7	transcription factor cys6 protein
T9765	NH	469	–	836	–	336	–	**16.3**	0.4	1.7	c6 transcription factor
T10202	Bcin12g02660.1	1341	6.4E-115	–	–	1058	2.7E-108	**50.3**	–	5.8	fungal specific transcription factor protein
T63	Bcin05g06170.1	–	–	2572	1.2E-93	1640	4.6E-93	–	4.0	**46.6**	transcription factor c2h2
T9641	NH	1903	–	202	–	–	–	**2.3**	0.2	–	camk protein kinase
T9481	Bcin05g04260.1	3343	0.0E + 00	670	1.7E-83	218	2.0E-30	**6.9**	0.8	0.3	serine threonine protein kinase
T321	Bcin10g03050.1	1327	0.0E + 00	1586	0.0E + 00	–	–	4.6	**43.5**	–	protein kinase-like protein
T312	Bcin14g02050.1	2114	0.0E + 00	1914	0.0E + 00	1770	0.0E + 00	7.0	**64.5**	4.9	regulator of g protein
T6048	Bcin03g09060.1	909	6.6E-34	560	1.4E-25	1458	1.3E-25	0.7	0.6	**6.1**	tetratricopeptide-like helical protein
T2642	Bcin11g05000.1	670	3.5E-19	512	1.6E-07	1419	4.0E-49	0.7	1.2	**131.2**	elongation factor 2 kinase
T9894	Bcin10g02660.1	1190	5.2E-143	967	7.3E-146	5797	1.6E-130	**199.6**	1.8	11.4	MSS4-like protein
Membrane proteins and transport											
T10087	NH	1497	–	–	–	–	–	**1.7**	–	–	related to antibiotic resistance protein
T9642	Bcin08g01830.1	1939	0.0E + 00	3904	2.0E-177	1626	0.0E + 00	**178.8**	13.0	13.2	MFS transporter
T9594	Bcin09g01740.1	2209	0.0E + 00	2369	0.0E + 00	1175	0.0E + 00	**18.7**	1.6	1.5	MFS drug efflux pump
T9657	Bcin12g01880.1	2434	0.0E + 00	577	1.9E-114	949	4.1E-158	**13.9**	1.0	1.6	MFS transporter
T10002	Bcin14g00580.1	2068	0.0E + 00	–	–	–	–	**5.2**	–	–	MFS multidrug transporter protein
T10106	NH	1913	–	–	–	–	–	**1.0**	–	–	MFS general substrate transporter
T9643	Bcin06g00130.1	2760	0.0E + 00	3266	0.0E + 00	1839	0.0E + 00	**114.9**	8.5	4.9	OPT oligopeptide transporter
T9629	Bcin10g00330.1	3305	0.0E + 00	1507	0.0E + 00	697	1.9E-144	**24.7**	2.0	0.7	potassium transporter protein
T10105	NH	1321	–	–	–	–	–	**11.7**	–	–	transmembrane protein
T9893	NH	1461	–	450	–	–	–	**84.0**	0.7	–	integral membrane protein
T9288	Bcin14g00590.1	1951	1.3E-117	–	–	–	–	**10.6**	–	–	putative integral membrane protein
T9835	Bcin12g05240.1	5309	0.0E + 00	508	8.5E-57	–	–	**8.7**	0.2	–	Tat pathway signal sequence protein
T323	Bcin03g01970.1	501	4.5E-07	2097	5.9E-09	–	–	1.1	**10.5**	–	DUF3328 domain containing protein
Secondary metabolite biosynthesis											
T10081	NH	1907	–	–	–	–	–	**1.9**	–	–	nrps-like enzyme protein
T10004	Bcin14g00600.1	5346	0.0E + 00	–	–	–	–	**0.9**	–	–	polyketide synthase protein
T8874	Bcin12g04980.1	7211	0.0E + 00	2477	0.0E + 00	5304	0.0E + 00	7.9	2.3	**64.2**	nonribosomal peptide synthase -like protein
T10094	NH	2132	–	–	–	–	–	**2.6**	–	–	bifunctional solanapyrone synthase
T6684	NH	1552	–	1450	–	1789	1.1E-21	0.8	0.6	**43.2**	bifunctional solanapyrone synthase
T10344	Bcin03g00060.1	–	–	–	–	2083	0.0E + 00	–	–	**23.2**	phenylacetate-CoA ligase paaK
T253	Bcin07g00950.1	1493	4.7E-68	1476	5.8E-68	1474	4.2E-66	15.4	**185.9**	15.9	short-chain dehydrogenase
T9627	Bcin03g01620.1	1643	0.0E + 00	1233	0.0E + 00	1240	0.0E + 00	**213.7**	16.8	15.2	nad dependent epimerase protein
T9913	NH	1385	–	461	–	–	–	**154.2**	0.8	–	cinnamoyl-CoA reductase
T10102	NH	835	–	–	–	–	–	**6.1**	–	–	beta-lactamase
T10099	NH	2921	–	–	–	–	–	**4.1**	–	–	beta-lactamase/transpeptidase-like protein
T9539	Bcin11g02630.1	1522	1.2E-16	1481	2.7E-16	1398	1.8E-15	**27.4**	2.9	2.9	phytanoyl-CoA dioxygenase family protein
T9957	Bcin07g07120.1	584	7.3E-90	–	–	–	–	**0.7**	–	–	enoyl-hydratase isomerase family protein
T10360	Bcin03g00080.1	–	–	–	–	1342	4.2E-117	–	–	**34.2**	taurine catabolism dioxygenase
T10358	Bcin03g00090.1	–	–	–	–	2115	0.0E + 00	–	–	**43.2**	taurine catabolism dioxygenase TauD
Oxidation-reduction processes											
T9579	Bcin15g04780.1	2025	0.0E + 00	2008	0.0E + 00	1869	0.0E + 00	**188.6**	17.4	20.4	cytochrome P450 monooxygenase
T7866	Bcin12g04940.1	4816	0.0E + 00	5506	0.0E + 00	1741	0.0E + 00	8.3	4.8	**144.9**	cytochrome P450 monooxygenase GliC
T271	Bcin12g06400.1	2729	1.7E-89	1723	3.7E-89	2006	4.1E-84	2.3	**26.9**	3.0	benzoate 4-monooxygenase cyt P450
T151	Bin07g05430.1	–	–	1917	0.0E + 00	1917	0.0E + 00	–	**91.9**	8.0	cytochrome P450 monooxygenase
T9563	Bcin11g00830.1	2364	0.0E + 00	2348	0.0E + 00	2064	0.0E + 00	**89.5**	9.0	2.5	flavin-binding monooxygenase
T9787	Bcin08g04930.1	3159	0.0E + 00	2405	0.0E + 00	6429	0.0E + 00	**51.2**	1.8	4.3	flavin-binding monooxygenase-like protein
T9590	NH	1899	–	246	–	241	–	**3.3**	0.3	0.3	fad linked oxidase domain protein
T9873	NH	2044	–	732	–	254	–	**28.3**	0.3	0.6	choline dehydrogenase/alcohol oxidase
T10204	Bcin09g04870.1	5827	4.5E-04	–	–	1888	3.2E-04	**25.7**	–	2.7	SDH assembly factor mitochondrial
T9693	Bcin12g01060.1	2089	0.0E + 00	894	1.3E-138	1435	0.0E + 00	**86.5**	5.2	9.5	indoleamine 2,3-dioxygenase beta type
T10270	Bcin07g03220.1	–	–	728	6.9E-15	–	–	–	**2.2**	–	carbonic anhydrase
T212	Bcin16g02850.1	2962	0.0E + 00	3150	0.0E + 00	2336	0.0E + 00	3.0	**46.5**	4.1	fad binding domain-containing protein
T9868	Bcin10g04440.1	1193	5.0E-136	–	–	–	–	**262.5**	–	–	SDR family protein
T10221	Bcin14g00490.1	–	–	1246	4.2E-34	–	–	–	**8.0**	–	short-chain dehydrogenase
T9930	Bcin01g01090.1	203	3.0E-13	1607	7.4E-156	–		0.3	**3.1**	–	short-chain dehydrogenase
T3472	Bcin02g00280.1	1460	2.7E-133	1953	9.8E-157	1862	9.4E-158	1.0	1.4	**13.3**	fad binding domain-containing protein
T4552	Bcin03g00320.1	1989	0.0E + 00	290	–	1740	0.0E + 00	1.2	1.4	**64.0**	oxidase
T242	Bcin12g06180.1	2314	0.0E + 00	1495	0.0E + 00	1395	0.0E + 00	32.4	**405.7**	32.3	nitrilase/cyanide hydratase
T134	Bcin13g05670.1	1347	0.0E + 00	1329	0.0E + 00	1473	0.0E + 00	29.8	**698.6**	52.6	nitrilase/cyanide hydratase
T378	Bcin01g05790.1	3598	0.0E + 00	3508	0.0E + 00	2494	0.0E + 00	11.2	**91.1**	8.3	nitrite reductase
Nucleic acid modification and metabolism											
T9754	Bcin05g02630.1	1567	0.0E + 00	2003	0.0E + 00	2430	0.0E + 00	**113.2**	5.3	10.5	zinc ion binding protein
T10103	NH	2588	–	–	–	–	–	**131.5**	–	–	group II intron reverse transcriptase/maturase
T10111	NH	5711	–	–	–	2937	–	4.0	–	**80.5**	group II intron reverse transcriptase/maturase
T4409	BC1G_20011mito	510	–	214	–	4757	–	1.4	1.6	**13.2**	group II intron
T10367	Bcin12g04370.1	–	–	–	–	4858	5.3E-73	–	–	**29.4**	retrotransposon polyprotein (Ty3/Gypsy)
T10387	Bcin03g00140.1	–	–	–	–	5352	0.0E + 00	–	–	**48.2**	retrotransposon polyprotein (Ty3/Gypsy)
T10365	Bcin10g04500.1	–	–	–	–	9377	1.0E-53	–	–	**37.7**	retrotransposon polyprotein (Ty3/Gypsy)
T10350	Bcin13g03280.1	–	–	–	–	4443	3.9E-29	–	–	**625.5**	retrotransposon polyprotein (Ty1/Copia)
T10389	Bcin09g03840.1	–	–	274	6.8E-26	2191	1.4E-98	–	0.1	**4.3**	retrotransposon polyprotein (Ty1/Copia)
T3334	NH	–	–	–	–	737	–	–	–	**163.6**	RT from transposon X-element
T5170	Bcin16g02550.1	–	–	–	–	1065	0.0E + 00	–	–	**150.5**	transposase
T10352	Bcin14g04350.1	–	–	–	–	2493	5.2E-15	–	–	**10.3**	chromo domain containing protein
T6826	Bcin10g02210.1	–	–	–	–	802	1.1E-04	–	–	**261.0**	chromo domain containing protein
T91	NH	–	–	4405	–	–	–	–	**6.4**	–	probable dna polymerase
T10215	Bcin16g04490.1	–	–	2149	2.4E-55	–	–	–	**1.5**	–	ribonuclease iii protein
T7829	Bcin03g00050.1	–	–	–	–	1759	0.0E + 00	–	–	**60.5**	ribonuclease H-like protein
T10342	Bcin13g01670.1	–	–	–	–	1939	1.3E-96	–	–	**3.2**	trna splicing endonuclease subunit protein
Miscellaneous											
T9817	Bcin12g06170.1	1248	1.4E-122	1658	1.5E-117	607	3.5E-58	**241.3**	6.5	1.0	allergen protein
T10319	Bcin13g01080.1	–	–	–	–	1891	3.6E-56	–	–	**3.7**	similar to DUF946 domain-containing protein
T10320	Bcin04g06320.1	–	–	–	–	1682	4.3E-04	–	–	**3.3**	putative heterokaryon incompatibility protein
T9635	NH	4825	–	682	–	331	–	**11.6**	0.9	1.1	HET-domain-containing protein
T326	Bcin04g00810.1	–	–	1446	0.0E + 00	–	–	–	**38.2**	–	ankyrin repeat protein
T357	Bcin13g01520.1	2371	5.4E-91	1623	3.7E-144	586	4.1E-94	1.4	**11.8**	1.1	ankyrin repeat-containing protein
T10346	Bcin01g11420.1	–	–	–	–	1019	1.2E-19	–	–	**1.8**	glutathione S-transferase protein
T10278	Bcin07g01490.1	–	–	1982	9.3E-12	395	–	–	**2.5**	–	AAA family ATPase
T9525	Bcin07g05160.1	6988	1.7E-119	595	1.1E-45	–	–	**3.3**	0.4	–	HSP mitochondrial precursor protein
T9961	Bcin06g06170.1	705	1.4E-20	–	–	–	–	**0.7**	–	–	MmgE/PrpD family protein
T9939	Bcin01g10190.1	438	7.6E-05	–	–	–	–	**0.7**	–	–	BTB/POZ domain-containing protein
T9988	Bcin10g02020.1	2167	3.1E-51	–	–	–	–	**4.7**	–	–	BTB/POZ domain-containing protein
T9546	NH	2010	–	430	–	–	–	**3.2**	0.3	–	SET domain-containing protein
T256	NH	1637	–	2277	–	537	–	2.2	**26.6**	0.7	tldc domain

^¥^ NH = No homologs in the *B. cinerea* proteome. Over-expressed transcripts are highlighted in bold

#### Hydrolytic and carbohydrate-active enzymes

Twelve DETs encoding hydrolytic or carbohydrate-active enzymes were over-expressed in MFRC, including glycoside hydrolases (GHs) such as a β-1,3-glucan binding protein of the GH16 family (T9687; FC = 11.4–16.2); two DETs (T9618 and T10205; FC ≥ 10), only partially reconstructed in MLAX and MFRG, encode respectively an α-L-arabinofuranosidase (GH51) and a chitin-binding type 1 protein (GH18) with a homolog in *Macrophomina phaseolina* (E-value = 4e-63; id = 58%); and the partial sequence (T10086) of a putative GH65 family protein assembled only in MFRC. Full-length or partial DETs annotated as α/β hydrolases were over-expressed (T9625, T9489 and T9732) or uniquely assembled (T10098) in MFRC. The protein encoded by T9489 (FC = 8.3, with no homologs in MFRG) contains conserved domains and exhibits some sequence similarity (E-value≥4e-35; id = 33%) to carboxylesterases and lipases from fungi, but not in *Botrytis* and *Sclerotinia* spp.. A galactose oxidase precursor protein (T9679; FC = 12.2–15.8) and a pectate lyase (T9581; FC = 10.4–13.4) were also detected. T10089, with no orthologs in MLAX and MFRG, showed similarities to a hypothetical protein of *S. sclerotiorum* and to amidohydrolase 2 of *M. phaseolina* (E-value = 4e-07; id = 46%) and other fungi, but not *B. cinerea*.

Nine DETs of the same enzymatic group were over-expressed in MLAX including a putative ß-1,6-galactanase protein (T309; FC = 9.7–19.4), member of GH5 family. Two additional GHs (FC = 8.0–11.8) were a putative xyloglucan-specific endo-ß-1,4-glucanase (GH12; T288) and an α-1,2-mannosidase (GH92; T310). A fungal phospholipase B-like domain was identified in T163 (FC = 8.5–20.2). α/β hydrolases included a prolyl aminopeptidase (T181; FC = 16.3–18.3), as well as two additional DETs (FC = 10.3–64.1) encoding a pectin methylesterase (T99) and laccase 2 (T53). Finally, the hydroxyacylglutathione hydrolase T96 (FC = 30.7–32.8) and a putative extracellular intradiol dioxygenase (T240; FC = 8.6–12.5) were among DETs identified in MLAX.

Four DETs were over-expressed in MFRG, including T7115 (FC = 9.0–12.8) encoding a putative α-amylase (GH13 family), and T6223 (FC = 18.8–22.1) encoding a ß-mannosidase B-like protein (GH2 family). Moreover, we detected a carbohydrate esterase family 12 protein (T8110; FC = 12.3–24.9), involved in degradation of pectin in cell wall, and a cellobiose dehydrogenase (CDH) (T9172; FC = 9.2–43.8), involved in cellulose degradation, having homologs (E-value = 0; id≥79%) in *S. borealis* and *S. sclerotiorum* but not in *B. cinerea*.

#### Effector and secreted proteins

Three DETs were over-expressed in MFRC. A putative protease inhibitor (T9637; FC = 12.9–39.3), containing a phosphatidylethanolamine-binding protein (PEBP) domain, was found. The full-length T9520 (FC = 9.0–11.3) encodes a protein homologous to RHS (rearrangement hotspot)- and YD (tyrosine-aspartate)-repeat containing proteins from several fungi (E-value = 0; id up to 74%), including *S. borealis* and *B. cinerea* and, with lower similarity (E-value = 0; id = 32–35%), to the type IV secretion protein RHS of *Pseudomonas* spp.. T9914 (FC = 10.1–202.0) encodes a secreted trypsin-like serine protease with homologs in the closely related species *B. cinerea*, *S. sclerotiorum*, *S. borealis* (E-value≤4e-141; id≥83%), as well as in other phytopathogenic fungi (e.g., *Verticillium*, *Fusarium* and *Alternaria* spp.) and biocontrol agents (e.g., *Trichoderma* and *Metarhizium* spp.) (E-values≤1e-57; id≥54%).

In MLAX we found a secreted pepsin-like protein (T144; FC = 15.6–22.3) with a conserved domain for aspergillopepsin-like aspartic proteases and homologs in *B. cinerea* (BcAP7), *S. borealis* and other fungi (E-value≤4e-93; id≥67%).

#### Morphogenesis and development

Six DETs were significantly over-expressed in MFRC. They included a developmental-specific protein (T9903; FC = 32.8–148.4), a putative fatty acid elongase (FEN1) of the GNS1/SUR4 family like protein (T9722; FC = 10.1–19.1), and a linoleate 8R-dioxygenase like protein (T9480; FC = 8.1–14.5) having homology (E-values = 0; id ≥46) with psi (producing precocious sexual inducer)-producing oxygenase A (PpoA) from *Rhynchosporium*, *Penicillium* and *Aspergillus* spp.. Two putative cell surface proteins were encoded by T9697 (FC = 16.0–19.9) and T9572 (FC = 9.2–10.0) having homology (E-value≤8e-71; id≥60%) with the *Blumeria graminis* f.sp. *hordei* gEgh16 and Egh16H, the *M. grisea* GAS1 (gEgh16 homologs expressed in Appressorium Stage) and MAS3 (Magnaporthe appressoria specific), and the *Colletotrichum* CAS1 (appressorium specific protein). T9843 (FC = 15.8–58.4) encodes a protein having similarities (E-value = 0; id = 34–41%) with fungal cell wall SED1 proteins.

A putative glycosylphosphatidylinositol-anchored protein (GPI-AP) was significantly over-expressed in MLAX (T205; FC = 15.2–33.5).

#### Regulation and signalling

The DETs over-expressed in MFRC included two transcription factors containing a N-terminal GAL4-like (Zn_2_Cys_6_) DNA-binding domain (GAL4 TF), T9760 (FC = 8.2–21.4) and T9765 (FC = 9.6–40.8). T10202 (FC = 8.7), with no orthologs in MLAX, showed homology (E-value = 2e-119; id = 65%) with a *B. cinerea* putative fungal specific TF. Two putative protein kinases (T9641 and T9481) were over-expressed in MFRC (FC = 8.6–23.0) as well as a putative MSS4 (mammalian suppressor of Sec4) like protein (T9894; FC = 17.5–110.9).

A regulator of G protein signalling (RGS) (T312) was over-expressed in MLAX (FC = 9.2–13.2) as well as a protein kinase-like sequence (T321; FC = 9.5), with no orthologous in MFRG, showing homology (E-value≥6e-84; id = 38%) with phosphotransferases and aminoglycoside phosphotransferases of *Aspergillus* and *Penicillium* spp..

A Zn finger C2H2 TF (T63) was over-expressed in MFRG (FC = 11.7), with no orthologs in MFRC. A MFRG elongation factor-2 kinase (eEF-2) (T2642; FC = 109.3–187.4) was fragmented in MFRC and MLAX. T6048 (FC = 8.7–10.2) encodes a putative protein with homology (E-value = 6e-09; id = 27%) with a tetratricopeptide (TPR)-like helical protein of *Eutypa lata*.

#### Membrane proteins and transport

Twelve DETs were over-expressed or unique in MFRC. T10087 encoding a putative major facilitator superfamily (MFS) transporter related to antibiotic resistance proteins from *Fusarium* spp. (E-value≥1e-65; id up to 50%), with no homologs in *Botrytis* and *Sclerotinia* spp., was reconstructed only in MFRC. The predicted protein contains domains conserved for MFSs and bacterial Bcr/CflA transporters, which are responsible for drug and antibiotic resistance [24]. Additional MFSs were over-expressed (T9642, T9594 and T9657) or exclusive (T10106 and T10002). T9642 (FC = 13.5–13.8) encodes a MFS protein containing a 4-hydroxyphenylacetate permease domain HpaX, with homology to a *B. cinerea* high-affinity nicotinic acid transporter (E-value = 0.0; id = 90%). DETs included an oligopeptide transporter of the OPT family (T9643) and a potassium uptake protein (T9629) (FC = 12.4–35.3). T10105, with no orthologs in MLAX and MFRG, encodes a predicted transmembrane protein, like TLC domain-containing fungal proteins, that may possess multiple functions such as lipid trafficking, metabolism, or sensing. T9893 (FC = 120.0), partially assembled in MLAX, shows homology with a hypothetical protein of *Valsa mali* (E-value = 3e-101; id = 44%) and integral membrane proteins of *Aspergillus* and other fungi (E-values≤6e-57; id = 37–40%), with no orthologs in *Botrytis* and *Sclerotinia* spp.. T9835 (FC = 43.5), fragmented in MLAX, encodes a putative twin-arginine translocation (Tat) pathway signal sequence protein, with homologs in various fungal species (E-value = 0.0; id up to 59%).

T323 encoding a DUF3328 domain-containing protein, over-expressed in MLAX (FC = 9.5) and partially reconstructed in MFRC, also showed similarity with a Tat pathway signal sequence protein of *Rosellinia necatrix* (E-value = 1e-50; id = 41%).

#### Secondary metabolite (SM) biosynthesis

T10081 was assembled only in MFRC and showed a low expression level; the encoded protein has a low similarity with a putative non-ribosomal peptide synthetase (NRPS)-like enzyme of *B. cinerea* (E-value = 9e-16; id = 52%). T10004, assembled only in MFRC, was annotated as polyketide synthase (PKS) homologous to BcPKS1 of *B. cinerea* (E-value = 0; id = 54%). Three additional DETs, exclusive for MFRC, have homologous genes (E-value≤3e-61; id≥53%) on the same *B. cinerea* chromosomic region (chromosome 14: 227,864–247,543) downstream the *BcPKS1* gene. They included, in the order: a serine hydrolase (FSH1) domain-containing protein (T9586); a MFS multidrug transporter (T10002); a hypothetical protein (T9288) showing similarities with fungal integral membrane proteins. T10094, assembled only in MFRC, containing D-lactate dehydrogenase and FAD binding domains, showed homology (E-value≤8e-65; id≥47%) with a bifunctional solanapyrone synthase of *V. mali* and putative FAD-binding oxidoreductases of several fungal species, such as *Talaromyces stipitatus* and *Alternaria alternata*, with no orthologs in *Botrytis* or *Sclerotinia* species. The DETs exclusive for MFRC included T10102 exhibiting amino acid similarities to *B. cinerea* β-lactamases (E-value≤2e-07, id = 34%) and T10099 encoding a putative beta-lactamase/transpeptidase-like protein, containing an AmpC (CubicO group peptidase, beta-lactamase class C family) domain, with homologs (E-value≤8e-34; id≥34%) in *Phialocephala scopiformis* and *R. necatrix*. T9627 was over-expressed in MFRC (FC = 12.7–14.1) and showed conserved domains for cinnamyl-alcohol dehydrogenase protein family and nucleoside-diphosphate-sugar epimerases. It shares homology with a *B. cinerea* 3-ß hydroxysteroid dehydrogenase/isomerase family protein (E-value = 0; id = 79%) and with putative dihydroflavonol-4-reductases from different fungi (best E-value = 5e-173; id up to 68%). In addition, T9913 encoding a putative cinnamoyl-CoA reductase and containing aldehyde reductase, extended (e) SDRs and NAD-dependent epimerase/dehydratase domains, was highly expressed in MFRC (FC = 192.8), fragmented in MLAX and not detected in MFRG. It has homologs in *S. borealis* (E-value = 0; id = 82%) but not in *B. cinerea*. A putative phytanoyl-CoA dioxygenase (PhyH) family protein (T9539), significantly over-expressed in MFRC (FC = 9.4), with homologs in *S. borealis*, *S. sclerotiorum* and *B. cinerea* (E-value = 0.0; id = 91–92%), shares homology with a clavaminate synthase-like protein of *Glarea lozoyensis* (E-value = 3e-152; id = 70%). A putative enoyl-hydratase isomerase family protein containing a transferase domain (T9957) is related to a fumigaclavine B *O*-acetyltransferase-like protein from *Acremonium chrysogenum* (E-value = 4e-34; id = 39%) and to trichothecene 3-*O*-acetyltransferases from *Taphrina deformans* and *Fusarium* spp. (E-values≤7e-08; id≥27%). A short-chain dehydrogenase/reductase displaying a clavulanic acid dehydrogenase, classical (c) SDR domain (T253) was significantly over-expressed in MLAX (FC = 11.7–12.1).

Orthologues of the BcNRPS1, coded T8874, were fragmented in MLAX and over-expressed in MFRG (FC = 8.1–27.9) like T6684 (FC = 54.0–72.0), which encodes a protein sharing homology with a FAD-binding-domain containing protein of *S. borealis* (E-value = 0; id = 89%) and bifunctional solanapyrone synthases from *Phialophora attae*, *Diplodia seriata* and *Penicillium chrysogenum* (best E-value = 1e-137; id up to 50%). Three transcripts related to secondary metabolism (T10344, T10360 and T10358) were assembled only in MFRG. T10344 encodes a conserved hypothetical protein containing a phenylacetate-coenzyme A (CoA) ligase domain, with homologs in *B. cinerea*, *S. borealis* and several *Penicillium* and *Aspergillus* species (E-value = 0; id up to 85%). Two taurine catabolism dioxygenases TauD/TdfA (T10360 and T10358), unique in MFRG and having homologs in *B. cinerea*, produce sulphite and aminoacetyldehyde from the sulphur-containing amino acid taurine that is used by several bacteria and fungi for growth under sulphate starvation [25]. T10360 exhibits also significant homology (best E-value = 0; id up to 90%) with proteins of *Penicillium*, *Aspergillus* and other fungi containing a clavaminic acid synthetase (CAS)-like domain.

#### Oxidation-reduction processes

A cytochrome P450 monooxygenase (T9579) over-expressed (FC = 9.2–10.8) in MFRC shows significant homology with isotrichodermin C-15 hydroxylases (CYP65A1) of *Rhynchosporium* spp. and *Phialocephala subalpine* (E-value = 0; id = 75%). Two flavin-binding monooxygenase-like proteins, T9563 and T9787, both having homologs in *S. borealis*, *B. cinerea* and *S. sclerotiorum* (E-value = 0; id≥77%), were significantly over-expressed in MFRC (FC = 9.9–35.8).

A benzoate 4-monooxygenase cytochrome P450 protein (T271), showing homology (best E-values = 2e-130; id up to 43%) with fungal trichodiene oxygenases, and a cytochrome P450 monooxygenase (T151) showing similarity to sterigmatocystin biosynthesis P450 monooxygenases of *Aspergillus niger* and *Colletotrichum chlorophyti* (best E-value = 4e-131; id up to 45%) were over-expressed in MLAX (FC = 9.0–11.5), like two nitrilase/cyanide hydratases (T242 and T134; FC = 12.5–23.4) and a putative nitrite reductase (T378; FC = 8.1–11.0).

A cytochrome P450 monooxygenase (T7866), having homologs in *S. sclerotiorum*, *B. cinerea* and *S. borealis* (E-value = 0; id≥89%) and showing similarity (E-value = 0) to trichodiene oxygenase cytochrome P450 of *Fusarium* spp. (id up to 55%) and GliC proteins of *Aspergillus* spp. (id up to 64%), was over-expressed in MFRG (FC = 17.5–30.2).

#### Nucleic acid modification and metabolism

A zinc ion binding protein (T9754) containing a WLM (WSS1-like metalloprotease) domain was over-expressed in MFRC (FC = 10.8–21.4); it is related to the DNA damage response proteins WSS1 involved in sister chromatid separation and segregation and shares homology with *B. cinerea* and *S. borealis* proteins (e-values = 0; id≥77%). In addition, a group II intron reverse transcriptase/maturase (T10103), with homologs in *Neurospora intermedia* (E-value = 1e-40; id = 32%) and other fungi but not *Sclerotiniaceae*, was uniquely assembled in MFRC.

A mitochondrial DNA polymerase type B protein (T91) was uniquely assembled in MLAX, as well as a ribonuclease III (RNAse III) protein (T10215).

A mitochondrial group II intron encoding a multifunctional protein, belonging to the LAGLIDADG family of homing endonucleases (T4409) was assembled only in MFRG while short fragments were reconstructed in MFRC and MLAX. A group II intron reverse transcriptase/maturase (T10111), over-expressed in MFRG (FC = 20.1) and assembled in MFRC, has a homolog in *Juglanconis oblonga* (E-value = 0; id = 52%). Several transcripts related to retroelements were generally identified as unique in MFRG. In detail, T10367 and T10387 encode polyproteins with conserved domains of reverse transcriptase (RT) and ribonuclease H (RNase H) enzymes from long terminal repeats (LTRs) retroelements of the Ty3/Gypsy family, sharing homology with proteins from *B. cinerea* and other fungi (E-values = 0; id≥46%). Related to the same family is a polyprotein containing RT, RNase H and retropepsin domains (T10365) with homologs in *S. sclerotiorum* (e-value = 0: id up to 62%). T10350 encodes a polyprotein with RT and RNase H domains from LTRs retroelements of the Ty1/Copia family with a homolog in *S. borealis* (E-value = 7e-119; id = 66%). T10389 encodes a retrotransposon protein with a gag-polypeptide of LTRs Copia-like domain, with a homolog in *B. cinerea* (E-value = 1e-97; id = 62%). Both LTRs Copia-like retrotransposons showed similarity to retrovirus-related Pol polyproteins from the plant transposon TNT 1–94 (best E-value = 1e-75; id up to 37%). T3334 is a partial sequence of a putative non-LTRs retrotransposon having homology with RNA-directed DNA polymerase from fungal transposon X-elements (E-value = 2e-77; id = 63%) of the Long INterspersed Elements (LINE) class. T5170 encodes a transposase of the DDE superfamily endonuclease with homologs in *B. cinerea* and *S. sclerotiorum* (E-value = 0; id up to 99%). Two chromo (CHRromatin Organisation MOdifier) domain containing proteins were identified: T10352 with similarity to a putative gag polymerase env protein of *B. cinerea* (E-value = 4e-27; id = 72%); T6826 with similarity to truncated Pol (E-value = 3e-43; id = 85%) and BRTN (E-value = 2e-29; id = 63%) from Boty-like retrotransposons of *B. cinerea*. A putative RNA exoribonuclease 3 (REX3) (T7829) with a homolog in *B. cinerea* (E-value = 0; id = 80%), contains a 3′-5′ exonuclease domain belonging to the DEDDh protein superfamily.

#### Miscellaneous

Among DETs over-expressed in MFRC we found an allergen protein (T9817; FC = 37.1–241.3) sharing homology with a peptide transport protein of *Aureobasidium pullulans* (E-value = 1e-73; id = 65%) and a dehydrin-like protein 2b of *A. alternata* (E-value = 1e-59; id = 57%). T9635 encode a HET-domain-containing protein fully reconstructed in MFRC and fragmented in MLAX and MFRG. T9525, encoding a putative heat shock protein (HSP) mitochondrial precursor with homologs in *S. borealis*, *S. sclerotiorum* and *B. cinerea* (E-value≤1e-111; id≥50%), was assembled in MFRC, fragmented in MLAX and absent in MFRG.

T326, uniquely assembled in MLAX, and T357, over-expressed (FC = 8.4–10.7), encode putative ankyrin repeat proteins. T10278, fully assembled in MLAX and partially in MFRG, encodes a putative ATPase of the AAA family.

T10319, unique in MFRG, showed homology with a putative DUF946 domain-containing *B. cinerea* protein (E-value = 2e-54; id = 49%) and the vacuolar sorting-associated protein 62 (VPS62) of *Ophiocordyceps sinensis* (E-value = 1e-46; id = 45%). T10320 was assembled only in MFRG and showed homology with HET-domain-containing proteins (best E-value = 0; id up to 73%). T10346 assembled only in MFRG encodes a protein with homology to the *B. cinerea* glutathione S-transferase BcGST22 (E-value = 1.2e-19; id = 45%) likely involved in detoxification of xenobiotics.

## Discussion

The complete transcriptomes of MFRC, MLAX and MFRG were reconstructed and annotated. Comparative analyses among orthologous transcripts revealed 65 transcripts over-expressed (FC≥8 and FDR≤0.05) or unique for MFRC, 30 in MLAX and 31 in MFRG.

DETs encoding hydrolytic or carbohydrate-active enzymes were identified; nine were GHs, each belonging to a different family according to the Carbohydrate-Active Enzymes database (CAZy) classification (http://www.cazy.org/Glycoside-Hydrolases.html), which represent the largest and most diverse family of biopolymer-degrading enzymes [26].

The GHs over-expressed in MFRC included a β-1,3-glucan binding protein that in *Phanerochaete chrysosporium* is involved in the degradation of laminarin [27]; an α-L-arabinofuranosidase which is homolog to the *Magnaporthe oryzae* MoAbfB required for full virulence and inducing host defence responses [28]; and a chitin-binding type 1 protein. Effector proteins with chitin binding activity are directly involved in pathogenic processes in *Cladosporium fulvum*, *M. oryzae* and *Mycosphaerella graminicola* [29] and along with chitinases are up-regulated during *S. sclerotiorum* infection on *Brassica napus* [30]. Pizzuolo et al. [6] compared isolates of MFRC, MLAX and MFRG for pectolytic activity and their isoenzyme patterns and reported no substantial differences in the production of pectolytic enzymes (i.e. pectin methylesterase, polygalacturonase and pectin lyase) useful to differentiate the three species. In our study, two enzymes involved in plant cell wall breakdown were highly over-expressed in MFRC: a pectate lyase and a galactose oxidase, which is a secreted enzyme well characterized in *Fusarium* spp. catalysing the oxidation of D-galactose and other primary alcohols to aldehydes with concomitant reduction of molecular oxygen to hydrogen peroxide [31]. Moreover, two GAL4 TFs, over-expressed in MFRC, are positive regulators for galactose-induced genes in *S. cerevisiae* and for genes encoding enzymes degrading plant cell-wall polysaccharides in pathogenic fungi. The GAL4 TF encoded by the *AbPf2* (*Alternaria brassicicola* pathogenicity factor 2) gene is essential for the pathogenicity of the fungus [32]*.* Several α/β hydrolases, including proteins with carboxylesterase and lipase activities, and a putative amidohydrolase were over-expressed or uniquely assembled in MFRC. Amidohydrolases catalyse the hydrolysis of a wide range of substrates bearing amide or ester functional groups and include ß-lactamases, e.g. penicillin amidohydrolase involved in antibiotic metabolism [33].

The DETs over-expressed in MLAX included plant cell-wall degrading enzymes, such as a pectin methylesterase, a ß-1,6-galactanase protein (GH5), a xyloglucan-specific endo-ß-1,4-glucanase (GH12) and an α-1,2-mannosidase (GH92). Furthermore, other over-expressed enzymes were identified, such as a lysophospholipase, virulence factors in fungal pathogens [30], an hydroxyacylglutathione hydrolase, also known as glyoxalase II, involved in detoxification of methylglyoxal and related toxicants generated from the degradation of glycolytic products [34], and a putative extracellular intradiol dioxygenase belonging to an enzyme family catalysing ring cleavage of catecholate derivatives and likely involved in lignin breakdown [35].

GHs over-expressed in MFRG include a putative α-amylase (GH13) acting on α-glycosidic bonds and involved in lignocellulose saccharification processes [36] and a ß-mannosidase B-like protein (GH2), enzymes displaying different substrate specificity in *Aspergillus* spp. that likely reflect the diversity of their functions [37]. Two additional cell-wall degrading enzymes were identified, a carbohydrate esterase family 12 protein, acting on pectin, and a cellobiose dehydrogenase which plays a key role during pathogenesis in *Sclerotium* species [38] and in lignocellulose degradation by wood-rotting fungi [39].

A protease inhibitor containing a PEBP domain was over-expressed in MFRC. The yeast PEBP homologue TFS1 is supposed to be a bridge between cell signalling and intermediate metabolism in *Saccharomyces cerevisiae* [40]. Protease inhibitors are fungal effectors involved in plant-pathogen interactions; e.g., the inhibitor encoded by the *C. fulvum* avirulence gene *Avr2* is secreted during tomato infection and acts on papain-like cysteine proteases involved in plant defence [41]. We also identified in MFRC an RHS repeat protein. This class of proteins includes secreted bacterial insecticidal and nematocidal toxins and intercellular signalling proteins mediating bacterial-host or bacterial-bacterial interactions (e.g., [42]) A different class of the same family includes the Ss-RHS1 secretory protein from *S. sclerotiorum* that is related to sclerotial development, appressoria differentiation and virulence [43]. A trypsin-like serine protease was over-expressed in MFRC while a secreted pepsin-like aspartic proteases was over-expressed in MLAX. Trypsin-related enzymes are supposed to play a specific role in pathogenicity since they are the major proteases produced by plant-pathogenic *Verticillium* spp. whereas there are no reports of trypsins being secreted by saprophytes [44]. Secreted aspartic proteases, commonly known as acid proteases, are endopeptidases involved in functions related to nutrition and pathogenesis [45].

Among DETs related to fungal morphogenesis and development, we found over-expressed in MFRC a developmental-specific protein homolog to the *S. sclerotiorum* sclerotium-specific protein (Ssp1) detected in all stages of sclerotium and apothecium development [46]. A phenotypic comparison among the three *Monilinia* species described in a recent study by Villarino et al. [7] showed that sclerotia and stromata differentiation, affecting fungal survival under unfavourable conditions, was the most important factor distinguishing MFRC from the other two species. Moreover, MFRC is described as able to produce apothecia from pseudosclerotia in mummified fruit under both natural and in vitro conditions [47]; further functional studies are needed to determine the role of *Ssp1* homologous gene in pseudosclerotia development and evolution of *Monilinia* spp.. MFRC is also characterized by a faster colony growth and more abundant sporulation than MLAX and MFRG [5, 7]. In this study, MFRC showed over-expression of a fatty acid oxygenase with homology to PpoA proteins which are cyclooxygenase-like enzymes responsible for oxylipin production and regulate the development of conidiophores and cleistothecia and the production of toxic metabolites and degradative enzymes affecting fungal development and plant-pathogen interactions [48]. Other DETs were over-expressed in MFRC: a MSS4-like protein playing a role in cell polarization, polar tip extension and hyphal growth in *Neurospora crassa* [49]; a fatty acid elongase (FEN1) involved in sphingolipid biosynthesis and in modulation of resistance to amphothericin B in yeasts [50]; and the SED1 protein which is believed involved in resistance to lytic enzymes. Besides, two MFRC cell surface proteins are homologous to the *Blumeria graminis* f.sp. *hordei* gEgh16 and Egh16H, *M. grisea* GAS1 and MAS3, and *Colletotrichum* CAS1, all virulence factors essential for the establishment of plant-pathogen interactions [51].

DETs over-expressed in MLAX include a GPI anchored protein, important for cell wall synthesis and integrity in *N. crassa* [52] and implicated in virulence and *in planta* proliferation in *Fusarium graminearum* [53]; a regulator of G protein signalling which play key roles in upstream regulation of fundamental processes in fungi, including vegetative growth, sporulation, mycotoxin and pigment production, pathogenicity and mating [54]; two ankyrin repeat proteins, mediating protein-protein interactions; and a putative ATPase of the AAA family, a large and diverse group of enzymes inducing conformational changes in a wide range of proteins associated with cell-cycle regulation, proteolysis, organelle biogenesis and intracellular transport [55].

A Ca2+/calmodulin-dependent (CaMK) and a serine/threonine protein kinases were over-expressed in MFRC while a protein kinase-like over-expressed in MLAX shares homology with phosphotransferases responsible for antibiotic resistance in *Aspergillus* and *Penicillium* spp. [56].

A Zn finger C2H2 TF was over-expressed in MFRG. Such TFs are involved in pathogenicity, catabolite repression, acetamide regulation, differentiation of fruiting body, as well as stress responses and multidrug resistance in yeasts and human fungal pathogens. In *B. cinerea* they are involved in phytotoxin biosynthesis, secondary metabolism, carbohydrate metabolism, transport, virulence and detoxification mechanisms [57, 58]. MFRG also showed over-expression of TPR-like helical protein involved in stress and hormone signalling in plants [59].

The full-length transcript of an HSP mitochondrial precursor was reconstructed in MFRC. HSPs are involved in various biological processes and play crucial roles in morphogenetic changes, stress adaptation and antifungal resistance [60].

Several transmembrane transporters were over-expressed or exclusive for MFRC: MFS transporters that are involved, like the ATP-binding cassette (ABC) transporters, in multidrug resistance to natural and synthetic toxicants and playing a key role in plant pathogens as a shield against plant defence compounds during the pathogenesis and in fungicide resistance [61]; two flavin-binding monooxygenase-like proteins with CzcO conserved domain associated with the cation diffusion facilitator CzcD, which are involved in metal tolerance or resistance [62]; and a putative signal sequence protein of the Tat secretion pathway that serves to actively translocate fully folded proteins across membranes [63].

The most common SMs in fungi are polyketides, terpenoids and shikimic acid-derived compounds. PKSs and NRPSs are key enzymes in the biosynthesis of SMs containing highly conserved functional domains. The corresponding genes in fungal genomes are frequently co-localized and co-expressed with other genes coding for other enzymes involved in the same biosynthetic pathway [64]. Bifunctional solanapyrone synthases, over-expressed in both MFRC and MFRG, are involved in the biosynthesis of the polyketide-derived phytotoxins solanapyrones in *Alternaria solani*, *Ascochyta rabiei* and other fungi. Their phytotoxicity is well documented but their contribution to the pathogenicity has been questioned, while antifungal activity against microorganic competitors has been reported during saprotrophic but not parasitic growth [65]. β-lactamase transcripts were reconstructed in MFRC. Bacterial β-lactamases are known to confer resistance to β-lactam antibiotics, while the fungal homologs have been supposed to be responsible for the degradation of plant or microbial lactam compounds; for instance, a β-lactamase-containing gene cluster (*FDB1*) of *Fusarium verticillioides* confers resistance to lactam phytoanticipins [66]. A phenylacetate-CoA ligase was reconstructed in MFRG. Enzymes of this family are involved in the degradation pathway of aromatic compounds and in the biosynthesis of the β-lactam antibiotic penicillin G in *P. chrysogenum* [67]. In MFRC we also found a highly expressed putative cinnamoyl-CoA reductase, enzymes that in plants are involved in the phenylpropanoid biosynthetic pathway and have homologues in some bacteria and fungi although lacking the full pathway [68]. Several DETs are involved in the biosynthesis of clavulanic acid, a potent inhibitor of bacterial class A serine β-lactamases, with weak antibiotic activity. The clavaminic acid synthetase (CAS) and clavulanic acid dehydrogenase (CAD) are involved in its biosynthesis [69]. The PhyH protein identified in MFRC shares homology with a CAS-like protein of the human fungal pathogen *Glarea lozoyensis*; a putative CAD protein was over-expressed in MLAX; and the TauD unique in MFRG exhibits significant homology to CAS-domain-containing proteins of *Penicillium*, *Aspergillus* and other fungi. Moreover, a PhyH protein coded by the *thnG* gene from *Streptomyces cattleya* is involved in the biosynthesis of the β-lactam antibiotic thienamycin [70].

Several DETs were involved in mycotoxin metabolism. In MFRC a putative enoyl-hydratase isomerase containing a transferase domain showed similarity with the fumigaclavine B *O*-acetyltransferase involved in the biosynthesis of fumigaclavine C, an ergot alkaloid produced by fungi of the *Trichocomaceae* family [71] and with the trichothecene 3-*O*-acetyltransferases from *Fusarium* spp., which modify the trichothecene mycotoxin deoxynivalenol (DON) and reduce its toxicity [72], while a cytochrome P450 monooxygenase is related to isotrichodermin C-15 hydroxylases involved in the biosynthesis of trichothecenes [73]. DETs identified in MLAX included a benzoate 4-monooxygenase cytochrome P450 protein homologous to fungal trichodiene oxygenases, like the *Fusarium sporotrichioides* Tri4, involved in the trichothecene biosynthesis [74], and a cytochrome P450 monooxygenase showing similarity to enzymes essential for the synthesis of the polyketide-derived mycotoxin sterigmatocystin [75]. DETs identified in MFRG included a cytochrome P450 monooxygenase sharing similarity with enzymes that in *Fusarium* spp. and *Aspergillus* spp. are involved in the biosynthesis of the trichothecene and gliotoxin [76].

Differences in detoxification mechanisms were observed. The glutathione S-transferase assembled in MFRG is likely involved in detoxification of xenobiotics. Two nitrilases/cyanide hydratases were highly expressed in MLAX. Phytopathogenic fungi use nitrilases/cyanide hydratase to detoxify cyanide or cyanide by-products generated by plants, like amygdalin and prunasin produced by stone fruits which contribute to defence against herbivores and fungal pathogens [77].

Group II introns were detected in MFRC and MFRG. In bacterial genome and eukaryotic organelles, they are supposed to be an ancient class of ribozymes and mobile retroelements using intron-encoded reverse transcriptase, maturase and DNA endonuclease activities for site-specific insertion into homologous intron-less alleles [78]. Several transcripts related to LTR retroelements of the Ty3/Gypsy and the Ty1/Copia families were generally identified as unique in MFRG, revealing an active retrotransposition in the pathogen genome.

An RNAse III protein was uniquely assembled in MLAX. Eukaryotic RNase III or RNase III like enzymes, such as Dicer, are involved in RNAi (RNA interference) and miRNA (micro-RNA) gene silencing [79].

## Conclusions

In this study, we provide the first large-scale reconstruction and annotation of the transcriptomes of the phytopathogenic fungi MFRC, MLAX and MFRG which are the main causes of heavy yield losses on pome and stone fruits all around the world. The assembled transcriptomes included about 30,000 transcripts per species that were mostly complete, with low to moderate levels of duplication, fragmentation and estimated missing transcripts. About 50% of transcripts were functionally annotated. The transcriptomes of the three *Monilinia* species did not show any significant differences in the GO and OG cluster functional categories. Consequently, orthologous transcripts were identified in the three species on the base of the *B. cinerea* proteome and, additionally, more than 400 transcripts specific for at least one of the *Monilinia* species with no homologs in the reference were detected. A comparative analysis among orthologs in the three species based on transcript abundance revealed DETs over-expressed or exclusive for each of the three species. They were mostly associated with biopolymer-degrading enzymatic activities, detoxifying activity, secondary metabolism, such as biosynthesis and metabolism of xenobiotics, i.e. antibiotics and toxins, as well as processes affecting fungal morphogenesis and development, diversity and pathogen interactions with the host plants and the microbiotes.

Biopolymer-degrading enzymes, which are frequently pathogenicity factors, were more numerous in MFRC and MLAX than in MFRG. They included effectors, such as a chitin-binding protein, and enzymes involved in antibiotic metabolism in MFRC, and a methylglyoxal detoxifying enzyme in MLAX. Secreted proteins related to pathogenicity were detected, like a pepsin-like protein in MLAX and a trypsin-related enzyme in MFRC which displays also a putative protease inhibitor acting as effector during pathogenesis. These results are consistent with previous findings displaying less aggressiveness components of MFRG compared to MFRC and MLAX, in terms of lesions and incubation and latency period on artificially inoculated nectarine fruit at postharvest [7]. Moreover, MFRC was characterised by a very high expression of the developmental-specific protein Ssp1, cell surface proteins acting as virulence factors, enzymes responsible for oxylipin production and regulation of spore differentiation and plant-pathogen interactions, a MSS4-like protein playing a role in apical hyphal growth, numerous MFS transporters related to pathogenesis and multidrug resistance, enzymes involved in the biosynthesis of antimicrobials (solanapyrone, clavulanic acid) and mycotoxins, and β-lactamases involved in antimicrobial resistance. MLAX was characterised by enzymes responsible for antibiotic resistance, CAD enzymes involved in clavulanic acid biosynthesis, a P450 protein putatively involved in biosynthesis of mycotoxins, and cyanide hydratases detoxifying cyanogenic compounds which are typically produced by stone fruit trees. MFRG was characterised by a glutathione S-transferase involved in detoxification of xenobiotics, solanapyrone synthases, and enzymes involved in the biosynthesis of β-lactam antibiotics, clavulanic acid and gliotoxin-like mycotoxins. The fungus displayed numerous DETs related to transposable elements indicating an intense transposition activity in its genome.

The genes differentially expressed in the three pathogens play relevant roles in morphogenesis and development, diversity and pathogenesis and are worthwhile of further investigations since they might explain their different fitness. The data obtained represent new insights in the transcriptome analyses of the three species of *Monilinia* and provide a new and comprehensive genetic resource that will contribute to get deeper knowledge on the population biology, physiology and plant-pathogen interactions of these important phytopathogenic fungi which are essential for improving sustainable crop protection strategies.

## Additional file

Additional file 1: **Table S1.** Summary of sequencing data. **Table S2.** Numbers of mapping reads on the *Monilinia fructicola* (MFRC), *M. laxa* (MLAX) and *M. fructigena* (MFRG) assembled transcriptomes. (DOCX 51 kb)

## Abbreviations

CAD: clavulanic acid dehydrogenase
CAS: clavaminic acid synthetase
CoA: coenzyme A
DET: differentially expressed transcript
EggNog: Evolutionary genealogy of genes: Non-supervised Orthologous Groups
FC: Fold Change
FDR: False Discovery Rate
GH: glycoside hydrolase
GO: Gene Ontology
GPI: glycosylphosphatidylinositol
HSP: heat shock protein
LTR: long terminal repeat
MFRC: *Monilinia fructicola*
MFRG: *Monilinia fructigena*
MFS: major facilitator superfamily
MLAX: *Monilinia laxa*
MSS4: mammalian suppressor of Sec4
NRPS: non-ribosomal peptide synthetase
OG: orthologous group
ORF: Open Reading Frame;
PEBP: phosphatidylethanolamine-binding protein
PhyH: phytanoyl-CoA dioxygenase
PKS: polyketide synthase
RGS: regulator of G protein signalling
RHS: rearrangement hotspot
RNase: ribonuclease
RPKM: reads per Kb of transcript per million mapped reads
RT: reverse transcriptase
SM: secondary metabolites
Tat: twin-arginine translocation
TF: transcription factor
TPR: tetratricopeptide repeat
