# Supplementary material for: De novo assembly and comparative transcriptome analysis of Monilinia fructicola, Monilinia laxa and Monilinia fructigena, the causal agents of brown rot on stone fruits
Source: BMC Genomics. 2018 Jun 5;19:436. doi: 10.1186/s12864-018-4817-4 (PMC5987419; doi:10.1186/s12864-018-4817-4)
Supplement: Supplementary file 1 — Table S1. Summary of sequencing data. Table S2. Numbers of mapping reads on the Monilinia fructicola (MFRC), M. laxa (MLAX) and M. fructigena (MFRG) assembled transcriptomes. (DOCX 51 kb) [file 12864_2018_4817_MOESM1_ESM.docx]

**Table S1.** Summary of sequencing data.

| **Sample ID** | **Sequencing data (Gb)** | **Reads (N°)** | **Paired-end**  **fragments (N°)** | **Bases with QS≥30 (%)** |
| --- | --- | --- | --- | --- |
| Mfrc123 D | 1.432 | 15,565,456 | 7,782,728 | 92.60 |
| Mfrc123 L | 0.872 | 9,482,656 | 4,741,328 | 91.79 |
| Mfrc123 C | 1.126 | 12,244,416 | 6,122,208 | 92.71 |
| Mfrc78 D | 0.957 | 10,396,788 | 5,198,394 | 92.21 |
| Mfrc78 L | 0.928 | 10,086,096 | 5,043,048 | 91.86 |
| Mfrc78 C | 1.073 | 11,660,872 | 5,830,436 | 92.77 |
| Mlax316 D | 1.498 | 16,285,100 | 8,142,550 | 92.98 |
| Mlax316 L | 0.845 | 9,180,940 | 4,590,470 | 91.98 |
| Mlax316 C | 1.002 | 10,894,900 | 5,447,450 | 92.95 |
| Mlax297 D | 1.109 | 12,057,562 | 6,028,781 | 92.36 |
| Mlax297 L | 0.877 | 9,532,508 | 4,766,254 | 91.70 |
| Mlax297 C | 1.104 | 11,999,148 | 5,999,574 | 92.73 |
| Mfrg269 D | 1.357 | 14,752,110 | 7,376,055 | 92.46 |
| Mfrg269 L | 1.139 | 12,379,634 | 6,189,817 | 92.46 |
| Mfrg269 C | 0.857 | 9,317,972 | 4,658,986 | 92.73 |
| Mfrg344 D | 1.139 | 12,375,774 | 6,187,887 | 91.54 |
| Mfrg344 L | 1.129 | 12,273,260 | 6,136,630 | 92.13 |
| Mfrg344 C | 1.060 | 11,525,650 | 5,762,825 | 92.40 |

**Table S2.** Numbers of mapping reads on the *Monilinia fructicola* (MFRC), *M. laxa* (MLAX) and *M. fructigena* (MFRG) assembled transcriptomes.

| **Sample ID** | **Total paired reads** | **Reads mapped in pairs (%)** | **Reads mapped in broken pairs (%)** | **Unmapped reads (%)** |
| --- | --- | --- | --- | --- |
| Mfrc123 D | 15,302,864 | 12,939,922 (84.6) | 2,129,491 (13.9) | 233,451 (1.5) |
| Mfrc123 L | 9,287,724 | 8,363,342 (90.1) | 728,717 (7.9) | 195,665 (2.1) |
| Mfrc123 C | 12,013,768 | 10,548,306 (87.8) | 1,112,600 (9.3) | 352,862 (2.9) |
| Mfrc78 D | 10,199,066 | 9,403,004 (92.2) | 594,489 (5.8) | 201,573 (2.0) |
| Mfrc78 L | 9,879,694 | 8,865,956 (89.8) | 835,419 (8.5) | 177,319 (1.8) |
| Mfrc78 C | 11,488,744 | 10,224,292 (89.3) | 900,862 (7.9) | 323,590 (2.8) |
| Mlax316 D | 15,995,148 | 13,903,848 (86.9) | 1,812,357 (11.3) | 278,943 (1.7) |
| Mlax316 L | 8,987,272 | 8,070,648 (89.8) | 729,555 (8.1) | 187,069 (2.1) |
| Mlax316 C | 10,700,264 | 9,492,766 (88.7) | 912,023 (8.5) | 295,475 (2.8) |
| Mlax297 D | 11,841,434 | 10,179,180 (86.0) | 1,458,332 (12.3) | 203,922 (1.7) |
| Mlax297 L | 9,327,374 | 8,409,398 (90.2) | 746,050 (8.0) | 171,926 (1.8) |
| Mlax297 C | 11,783,956 | 10,271,402 (87.2) | 1,300,193 (11.0) | 212,361 (1.8) |
| Mfrg269 D | 14,536,228 | 12,706,518 (87.4) | 1,731,169 (11.9) | 98,541 (0.7) |
| Mfrg269 L | 12,158,444 | 11,258,538 (92.6) | 669,892 (5.5) | 230,016 (1.9) |
| Mfrg269 C | 9,154,232 | 8,258,398 (90.2) | 679,669 (7.4) | 216,165 (2.4) |
| Mfrg344 D | 12,012,540 | 10,043,836 (83.6) | 1,856,386 (15.5) | 112,318 (0.9) |
| Mfrg344 L | 12,052,462 | 10,116,498 (83.9) | 1,810,040 (15.0) | 125,924 (1.0) |
| Mfrg344 C | 11,250,654 | 10,016,408 (89.0) | 963,837 (8.6) | 270,409 (2.4) |
